# Proteomic and metabolomic analysis of serum in women infected with COVID-19 during late pregnancy

**DOI:** 10.3389/fimmu.2025.1589239

**Published:** 2025-06-11

**Authors:** Yi Zheng, Jinyu Ning, Jiang Zhu, Honglin Zhu, Zhihua She, Pei Cai

**Affiliations:** ^1^ Department of Pharmacy, Hunan Provincial Maternal and Child Health Care Hospital, Changsha, Hunan, China; ^2^ Department of Pathology, Xiangya Hospital, Central South University, Changsha, Hunan, China; ^3^ Rheumatology and Immunology Department, Xiangya Hospital, Central South University, Changsha, Hunan, China

**Keywords:** SARS-CoV-2, COVID-19, pregnant woman, proteomics, metabolomics

## Abstract

**Introduction:**

To investigate the alterations of serum proteins and metabolomics in women infected with severe acute respiratory syndrome coronavirus 2 (SARS-CoV-2) at the end of pregnancy and their potential effects on fetal development.

**Methods:**

The corona virus disease 2019 (COVID-19) group (n=31) included women in the third trimester diagnosed with SARS-CoV-2 infection and who delivered, while the control group (n=30) comprised uninfected women in the same gestational period. This study applied data-independent acquisition (DIA) proteomics and ultra-performance liquid chromatography coupled with quadrupole time-of-flight mass spectrometry (UPLC-Q-TOF-MS) metabolomics to analyze serum samples from two groups of full-term pregnant women. Serum samples in the control group were collected one week before delivery, while those in the COVID-19 group were collected within two days after the onset of fever. The differences between groups were compared by bioinformatics data analysis. For proteins and metabolites exhibiting a significant association with SARS-CoV-2, metabolic pathway enrichment was performed utilizing MetaboAnalyst 6.0, and the possible targets and pathways of SARS-CoV-2 infection in women in late pregnancy were plotted.

**Results:**

The incidence of cesarean section, postpartum reproductive tract infection, and fetal distress were significantly higher in the COVID-19 group compared to the control group. Differential proteomic analysis revealed the regulation of proteins such as SAA1, SAA2, IPO7, WDR19, and BAZ1A, which were involved in processes such as visual, skin and limb development. Metabolomics analysis revealed key altered metabolites, including 1-(7-methoxy-2-oxo-2H-chromen-8-yl)-3-methyl-2-oxobutylacetate, 5-(hydroxymethyl) -4-methoxy-2,5-dihydrofuran-2-one, and cyclocytidine, which were involved in the riboflavin metabolism, the phenylalanine, tyrosine and tryptophan biosynthesis, and the arginine biosynthesis. Integrative analysis of proteomic and metabolomic revealed significant disruptions in metabolic pathways, including arginine biosynthesis, steroid hormone biosynthesis, and fatty acid degradation.

**Conclusions:**

This study revealed the main proteomic and metabolic effects of SARS-CoV-2 infection on women in the third trimester of pregnancy. Our comprehensive omics data elucidating the molecular mechanisms underlying SARS-CoV-2 infection in women during late pregnancy. These findings offer novel insights and potential targets for future investigations into the impact of SARS-CoV-2 infection on maternal and infant health.

## Introduction

Since 2019, the severe acute respiratory syndrome coronavirus 2 (SARS-CoV-2) has spread worldwide, triggering a corona virus disease 2019 (COVID-19) pandemic. As of November 2024, the World Health Organization reports a cumulative total of 776 million confirmed COVID-19 cases and over 7.05 million deaths globally (1).The continuous variation of SARS-CoV-2 has led to the general vulnerability of the population to repeated infection. This persistent challenge has significantly strained global public health systems, and prompted the scientific research on further exploration of SARS-CoV-2 and its related diseases.

Pregnant women have been considered as a vulnerable group during the SARS-CoV-2 epidemic, owing to their special immunosuppression and immune tolerance status (2, 3). These alterations can increase the risk of immune response imbalances, rendering pregnant women more susceptible to severe viral infections. The mortality rate of COVID-19 in pregnant population is 11.3%, much higher than that in non-pregnant population (6.4%), and the risk of adverse pregnancy outcomes is significantly increased as well (4). Studies have shown that SARS-CoV-2 infection in pregnant women greatly increases the risk of serious pregnancy complications, including preeclampsia, HELLP syndrome, and ICU admission. Furthermore, the infection had detrimental effects on the fetus, leading to preterm birth, low birth weight, respiratory distress, and neurodevelopmental disorders (5). Therefore, it is crucial to explore the mechanism and the impact of SARS-CoV-2 on pregnant women and fetuses in order to formulate effective prevention and treatment strategies.

Studies have demonstrated that, compared with the normal group, patients with COVID-19 exhibit significant dysregulation in various apolipoproteins, including APOA1, APOA2, and APOH, as well as alterations in key pathways such as macrophage function, platelet degranulation, and complement system activation (6). Whole-proteome gene co-localization analysis revealed that ABO, OAS1, CD209, and FAS may serve as potential targets in COVID-19 (7).

Metabolomic analysis has also revealed significant changes in lipid metabolism in COVID-19 patients, with elevated levels of metabolites such as unsaturated fatty acids, lactic acid, and ketone bodies (8, 9). Roberts et al. (10) identified more than 20 differential metabolites between mild and severe COVID-19 cases through untargeted metabolomic analysis, primarily involving pyrimidine metabolism, tryptophan metabolism, and acylcarnitine metabolism. An increase in plasma kynurenine levels, accompanied by decreased levels of lysophosphatidylcholine and secondary bile acids, was also confirmed to be associated with COVID-19 progression (11). Although studies on metabolic abnormalities in individuals with COVID-19 are gradually increasing, research specifically focusing on pregnant women remains scarce. A recent meta-analysis (12) confirmed that the ratio of soluble fms-like tyrosine kinase-1 (sFlt-1) to placental growth factor (PlGF) was significantly higher in pregnant women with COVID-19 compared to healthy pregnancies. Torres et al. (13) also found that elevated serum sFlt-1 levels were associated with ICU admission, viral sepsis, and adverse maternal outcomes. In addition, previous studies have reported significantly increased levels of lysophospholipids, triglycerides, sphingolipids, and oxidized lipids in both maternal and umbilical cord plasma following SARS-CoV-2 infection (14).

Although substantial studies have explored the effects of SARS-CoV-2 on metabolism and protein expression in COVID-19 patients, the impact of SARS-CoV-2 on metabolic processes and protein expression in pregnant women and fetuses remains insufficiently explored. In particular, there is a lack of systematic analyses integrating metabolomics and proteomics, making it difficult to elucidate potential synergistic mechanisms. This study aims to further explore this area through a multi-omics approach and to address the current gaps in the literature.

Therefore, our study aims to explore the metabolic characteristics of women infected with SARS-CoV-2 at the end of pregnancy and its effects on fetal outcomes by combining proteomics and metabolomics techniques. We aim to offer innovative ideas and strategies for the prevention, diagnosis, and treatment of SARS-CoV-2 in pregnant women. Additionally, we seek to establish a scientific foundation for the development of personalized treatment and prevention approaches of SARS-CoV-2 infection.

## Materials and methods

### Research subjects and sample collection

A total of 61 full-term pregnant women in the acute phase of infection were recruited, and serum samples were collected. The sample size design was based on relevant literature (15–17), in which a sample size of 30–40 cases is typically sufficient to yield meaningful results. Furthermore, through pre-study power analysis (14), we calculated that at least 24 samples per group were required, and a total sample size of 61 is adequate to meet the requirements of an exploratory study. 31 participants with SARS-CoV-2 infection were selected as the observation group (COVID-19), and 30 healthy women were selected as the control group (Control).

In this study, pregnant women in both the COVID-19 group and the control group were enrolled approximately one week before delivery. Serum samples from the control group were collected during this period, whereas serum samples from the COVID-19 group were collected within two days after the onset of fever to capture the acute phase of infection. The inclusion criteria were as follows: (1) Patients in the COVID-19 group met the diagnostic criteria specified in the ‘Prevention and control of COVID-19 (9th edition)’, and obtain specimens within two days of the onset of fever caused by SARS-CoV-2 infection; (2) Gestational age ≥ 37 weeks. Exclusion criteria included severe pregnancy complications, such as gestational hypertension, poor glycemic control of gestational diabetes mellitus, and intrahepatic cholestasis of pregnancy. In addition, conditions that might affect fetal growth and the intrauterine environment were excluded, including intrauterine growth restriction, amniotic fluid abnormalities (polyhydramnios or oligohydramnios), placental abruption, placental insufficiency, fetal congenital structural abnormalities, and multiple pregnancies.

All enrolled pregnant women were of Han ethnicity. There were no statistically significant differences between the two groups (P > 0.05) in demographic characteristics, including age, parity, place of residence (urban or rural areas), employment status, smoking history, alcohol consumption, and BMI (all above 26). In addition, no significant differences were observed in health status between the groups, including allergy history (such as penicillin or cephalosporin allergy). All participants were confirmed through prenatal screening to have no thyroid diseases, cardiovascular diseases, hepatic or renal diseases, genetic disorders, or chronic diseases (such as chronic hepatitis or tuberculosis). Therefore, the two groups were highly comparable. Multivariable regression analysis was performed to further control for potential confounding factors affecting the study outcomes. The study was conducted in accordance with the Declaration of Helsinki. This study was approved by the Medical Ethics Committee of Hunan Provincial Maternal and Child Health Care Hospital (Approval No. 2022017). Written informed consent for inclusion was obtained from each subject before enrolment in the study.

### Quantitative proteomics analysis of serum using DIA

BioRAD proteominer beads were mixed with serum samples and rotated for 2 h. The mixture was then centrifuged at 10,000 g for 5 min at 4°C, and the supernatant was discarded. The beads were washed with wash buffer, and 0.4 mL of 1% TFA was added to elute the proteins. This elution step was repeated twice. The collected supernatant was frozen and lyophilized to obtain dry protein powder. The protein precipitate was dissolved in protein lysis solution (8 M urea, 100 mM TEAB, pH 8.5), and 1M DTT was added for reduction at 56°C for 1 h. After incubation on ice for 2 min, IAM was added for alkylation in the dark at room temperature for 1 h. Protein concentrations were determined using the Bradford protein assay, and standard curves were plotted to calculate the concentrations. A 20 µg protein sample was separated by 12% SDS-PAGE, and the bands were analyzed after staining. Protein digestion was performed by adding proteolytic solution and trypsin, followed by incubation at 37°C for 4 h and subsequent overnight digestion. The pH was adjusted to less than 3 with formic acid, and the sample was centrifuged and desalted through a C18 column, followed by washed and lyophilized.

Mobile phase A (0.1% formic acid) and mobile phase B (80% acetonitrile, 0.1% formic acid) were prepared. The lyophilized powder was redissolved in mobile phase A, centrifuged at 14,000 g for 20 min at 4°C, and 200 ng of the supernatant was injected for analysis. Peptides were separated using a Vanquish Neo UHPLC system (Thermo Fisher Scientific, MA, USA) equipped with a C18 column (ES906, PepMapTM Neo UHPLC, 150 μm × 15 cm, 2 μm, Thermo Fisher Scientific, MA, USA). The elution gradient was as follows: 0-0.2 min, 4% B; 0.2 -0.3 min, 4% B; 0.3-0.4 min, 4%-8% B; 0.4-0.7 min, 8% B; 0.7-13.7 min, 8%-22.5% B; 13.7-20.6 min, 22.5%-35% B; 20.6–21 min, 35%-55% B; 21-21.5 min, 55%-99% B; and 21.5-23.8 min, 99% B. The flow rate of the mobile phase was maintained at 0.5 μL·min^-1^. Mass spectrometry was performed on a Orbitrap Astral mass spectrometer (Thermo Fisher Scientific, MA, USA) in data-independent acquisition (DIA) mode. The ion source was electrospray ionization, with a spray voltage of 1.9 kV and an ion transfer tube temperature of 290°C. The primary mass spectrometry scan range was m/z 380-980, with a resolution of 240,000 (at m/z 200). The secondary mass spectrometry scan range was m/z 150-2000, with a maximum injection time of 3 ms.

### Serum metabolomics analysis

The analysis was performed using a Vanquish UHPLC system (Thermo Fisher Scientific, MA, USA) equipped with a Hypersil Gold C18 column (100 mm × 2.1 mm, 1.9 μm, Thermo Fisher Scientific, MA, USA) and a Q ExactiveTM HF mass spectrometer (Thermo Fisher Scientific, MA, USA). The column temperature was maintained at 40°C, and the injection volume was set at 2 μL. The mobile phase consisted of 0.1% formic acid aqueous solution (A) and methanol (B), with a flow rate was set at 0.2 mL·min^-1^. Gradient elution was carried out as follows: 0-1.5 min, 2% B; 1.5–3 min, 2%-85% B; 3–10 min, 85%-100% B; 10-10.1 min, 100% B; 10.1–11 min, 100%-2% B; and 11–12 min, 2% B. The mass spectrometry analysis was conducted using an electrospray ionization ion source, with a spray voltage set to 3.5 kV. The sheath gas flow rate was maintained at 35 psi, and the auxiliary gas flow rate was set to 10 L·min^-1^. The ion transfer tube temperature was maintained at 320°C, and the ion introduction radiofrequency level was adjusted to 60 V. The auxiliary gas heater temperature was set to 350°C. MS/MS secondary scans were performed in data-dependent mode.

### Proteomics data processing and analysis

Raw data from the proteomics analysis were processed using DIA-NN (v1.8.1) with the human protein sequences from UniProt (https://www.uniprot.org/) as the library, which searched the species-specific protein library. Retention times were corrected using iRT standards. Peptide-spectrum matches (PSMs) and proteins with a confidence level above 99% were retained, and peptides and proteins with a false discovery rate (FDR) greater than 1% were removed to ensure data quality. The error rate of protein was set to ≤ 0.01, the error rate of peptide was ≤ 0.01, and the reliability of peptide was ≥ 99%. The Q-value cutoff value of precursor ion was set at 0.01. Statistical analysis of protein quantification results was performed using the Student’s t-test. Proteins exhibiting significant quantitative differences between the COVID-19 group and the control group (P < 0.05, fold change (FC) ≥ 2 or FC ≤ 0.5) were defined as differentially expressed proteins (DEP). The DEPs were further analyzed using the DEP package (v1.16.0) for identification, and gene set enrichment analysis (GSEA) was conducted using the R package clusterProfiler. The quality control analysis of the plasma proteomics data is presented in **Supplementary Figure S1**.

### Metabolomics data processing and analysis

The raw mass spectrometry data were processed to include peak area correction, peak alignment, peak extraction, and deconvolution. Molecular formulas were predicted based on molecular ion peaks and fragment ions, and metabolites were identified by comparison with the mzCloud (https://www.mzcloud.org/), mzVault, and Masslist databases. A matching similarity threshold of ≥0.8 was applied to ensure the reliability of metabolite identification. Metabolites were annotated using the KEGG database (https://www.genome.jp/kegg/pathway.html), HMDB database (http://www.hmdb.ca/metabolites), and LIPIDMaps database (http://www.lipidmaps.org/). Metabolic pathway enrichment analysis was performed using MetaboAnalyst 6.0 (https://www.metaboanalyst.ca/). MetaX software was used to perform principal component analysis (PCA) and partial least squares discriminant analysis (PLS-DA) after data conversion. The variable importance in projection (VIP) of each metabolite was calculated. Differential metabolites were identified based on the criteria of VIP > 1, P < 0.05 and FC ≥ 2 or FC ≤0.5.

### Integrated multi-omics analysis

To further integrate the proteomic and metabolomic datasets, we utilized the pathway-level module in MetaboAnalyst 6.0 (https://dev.metaboanalyst.ca/MetaboAnalyst/upload/JointUploadView.xhtml). A list of 152 significantly differentially expressed proteins with their corresponding FC values and 172 significantly altered metabolites with their FC values was uploaded. Pathway enrichment analysis was performed using the hypergeometric test, and the results were ranked based on the P-values and pathway impact scores. The most representative altered pathways were subsequently identified according to these rankings. The workflow of the integrated proteomic and metabolomic analysis is shown in **Supplementary Figure S2**.

### Statistical analysis

Statistical analysis was performed using SPSS 26.0 software (IBM, IL, USA). Normally distributed data are expressed as mean ± standard deviation and were analyzed using a t-test. Non-normally distributed data are presented as median (interquartile range) and analyzed by Wilcoxon rank sum test. Categorical data were analyzed by χ2 test. P < 0.05 indicates that the difference is statistically significant. Volcano plots and bubble plots were created using the R package ggplot2, and clustering heatmaps were generated using the R package Pheatmap.

## Results

### Clinical features of the control and COVID-19 groups

There were no significant differences in age, parity, or gestational age of delivery between the two groups. However, the number of pregnancies among pregnant women in the COVID-19 group was significantly higher than that in the control group (P 0.05) (**Table 1**). The successful rate of natural delivery in the COVID-19 group was significantly lower than that in the control group (P 0.05), while the incidence of postpartum reproductive tract infections and fetal distress during labor was significantly higher in the COVID-19 group (P 0.05) (**Table 2**).

**Table 1:** Comparison of general data between the control group and the COVID-19 group.

General data	Control (n=30)	COVID-19 (n=31)	Z value/T value/χ^2^ value	P value
Age (years)	29 (4)	29 (4)	z=0.036	0.971
Number of pregnancies (times)	1 (1)	2 (2)	z=2.792	0.005^**^
Parity (times)	1 (0)	1 (1)	z=0.249	0.804
Gestational age of delivery (weeks)	38 (2)	38 (1)	z=0.16	0.873
BMI (kg/m^2^)	26.55 ± 2.27	26.87 ± 3.16	t=0.13	0.897
Number of unemployed individuals	5	7	χ^2^ = 0.337	0.561
Number of individuals living in rural areas	9	6	χ^2^ = 0.943	0.334
Number of individuals with a history of alcohol abuse	0	0	–	–
Number of individuals with a smoking history	0	1	–	1.000
Number of individuals with an allergy history (%)	1	2	–	1.000

Continuous variables are presented as median (interquartile range) or mean ± standard deviation. Continuous variables were compared using the Mann-Whitney U test or t-test, and categorical variables were compared using the Chi-square test or Fisher’s exact test, as appropriate. ^**^P < 0.01 was considered statistically significant.

**Table 2:** Comparison of delivery mode, pregnancy complications and neonatal outcomes between the control and COVID-19 groups.

Indicators	Control (n=30)	COVID-19 (n=31)	χ^2^ value/ T value	P value
Cesarean section [numbers (%)]	9 (30)	18 (58.1)	χ^2^ = 4.867	0.027^*^
Postpartum reproductive tract infection [numbers (%)]	0	5 (16.1)	χ^2^ = 5.271	0.022^*^
Umbilical cord abnormality [numbers (%)]	6 (20)	9 (29)	χ^2^ = 0.671	0.412
Fetal distress [numbers (%)]	1 (3.3)	6 (19.4)	χ^2^ = 3.852	0.05^*^
Premature rupture of membranes [numbers (%)]	10 (33.3)	10 (32.3)	χ^2^ = 0.008	0.929
Adherent placenta [numbers (%)]	0	2 (6.5)	χ^2^ = 2.001	0.157
Macrosomia [numbers (%)]	1 (3.3)	1 (3.2)	χ^2^ = 0.001	0.981
Weight of infants (g)	3263 ± 406.4	3270 ± 347.1	t=0.075	0.941

^*^P < 0.05 was considered statistically significant.

In the late pregnancy, the COVID-19 group exhibited significantly lower levels of platelet count and neutrophil-to-lymphocyte ratio (NLR) compared to the control group (P 0.05), Conversely, the COVID-19 group had significantly higher levels of creatine kinase, myoglobin, and aspartate aminotransferase (AST) than the control group (P 0.05) (**Table 3**). The clinical characteristics and treatment of the COVID-19 group are presented (**Supplementary Table S1**).

**Table 3:** Comparison of clinical indicators between the control and COVID-19 groups.

Clinical indicators		Control (n=30)	COVID-19 (n=31)	T value/ Z value	P value
Blood routine	Leucocyte (×10^9^/L)	10.03±3.59	8.84±2.91	t=1.879	0.698
	Erythrocyte (×10^12^/L)	3.86 (0.58)	3.68 (0.67)	z=1.768	0.077
	Hemoglobin (g/L)	114.5 (14.75)	112 (13.25)	z=1.074	0.283
	Platelet (×10^9^/L)	216 (74.25)	174 (81)	z=2.979	0.003^**^
	Neutrophils ratio (%)	74.84±6.13	79.82±7.34	t=3.798	0.521
	Lymphocyte ratio (%)	18.17±5.39	12.43±6.33	t=4.743	0.952
	NLR (%)	4.34 (2.65)	2.84 (1.47)	z=4.875	<0.001^***^
Blood lipid	Triglyceride (mmol/L)	3.61±1.03	4.13±1.11	t=0.801	0.944
	Total cholesterol (mmol/L)	5.89±0.58	6.46±0.98	t=1.13	0.308
	HDL-C (mmol/L)	1.89±0.33	1.88±0.28	t=0.087	0.697
	LDL-C (mmol/L)	3.02±0.46	3.25±0.69	t=0.628	0.471
Myocardial enzyme	Creatine kinase (U/L)	31.4 (10.2)	64 (176.8)	z=2.113	0.035^*^
	Lactic dehydrogenase (U/L)	173 (26.5)	188.1 (65.8)	z=0.589	0.556
	Creatine kinase isoenzyme (U/L)	9.1 (4.8)	10.3 (9.1)	z=0.907	0.365
	Myoglobin (ng/mL)	13.6 (7.7)	24.1 (60.9)	z=2.219	0.026^*^
Liver function	Albumin (g/L)	35.89±2.72	34.51±4.07	t=0.973	0.445
	Globulin (g/L)	27.51±2.16	26.86±4.06	t=0.498	0.214
	Albumin/Globulin	1.31±0.13	1.28±0.20	t=0.398	0.315
	ALT (U/L)	9.7 (11.8)	10.4 (26.35)	z=0.898	0.369
	AST (U/L)	16.6 (8.52)	24.3 (24.5)	z=2.667	0.008^**^
	TBIL (μmol/L)	6.2 (4.05)	6.96 (5.69)	z=0.707	0.479
	DBIL (μmol/L)	2.6 (1.61)	2.68 (2.47)	z=0.844	0.399
Renal function	Creatinine (μmol/L)	54.33±6.34	51.94±10.23	t=0.582	0.213
	Uric acid (μmol/L)	304.25±54.61	324.55±67.05	t=0.702	0.806
	Urea (μmol/L)	3.45±0.74	2.9±1.28	t=1.086	0.051

^*^P < 0.05, ^**^P < 0.01, ^***^P < 0.001 was considered statistically significant.

NLR, neutrophil-to-lymphocyte ratio; HDL-C, high-density lipoprotein cholesterol; LDL-C, low-density lipoprotein cholesterol; ALT, alanine aminotransferase; AST, aspartate aminotransferase; TBIL, total bilirubin; DBIL, direct bilirubin.

### DIA quantitative proteomic analysis in the control and COVID-19 groups

**Figure 1:**
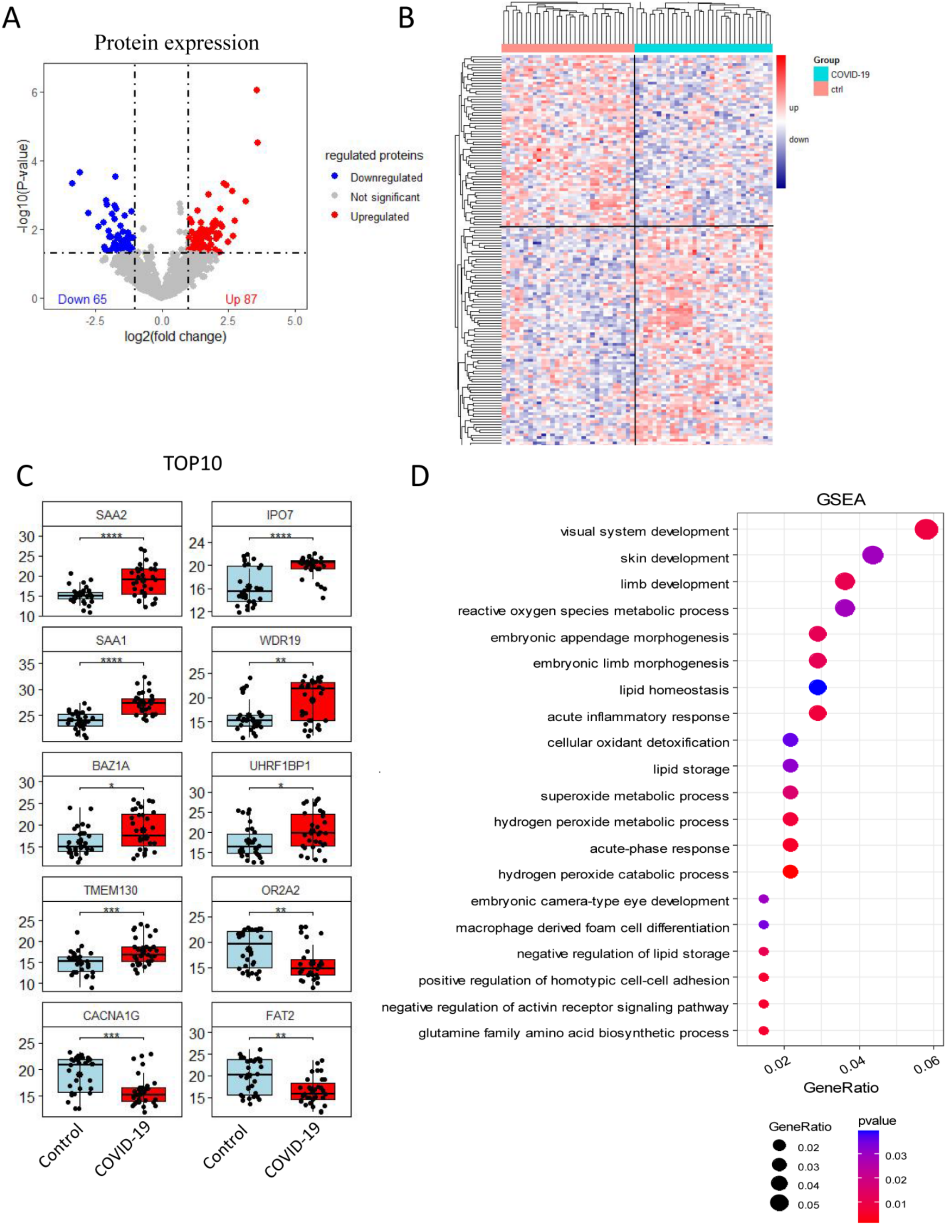
Quantitative proteomic analysis via DIA. **(A)** Volcano plot and **(B)** heat map showing, 152 differentially expressed proteins between the control and COVID-19 groups, with 87 up-regulated and 65 down-regulated proteins. **(C)** Cluster analysis results displaying the top 10 proteins with the most significant differential expression. **(D)** GSEA plot showed the potential regulatory physiological processes. The size of the circle indicates the number of enriched proteins, while the red color reflects the statistical significance of the enrichment.

A total of 152 differentially expressed proteins were identified through DIA quantitative proteomic analysis of serum from both the control and COVID-19 groups, with 87 proteins being up-regulated and 65 down-regulated (**Figures 1A, B**). The top 10 proteins with the most significant alteration included the up-regulated proteins SAA1, SAA2, IPO7, WDR19, BAZ1A, UHRF1BP1, and TMEM130 (P 0.05), and the down-regulated proteins OR2A2, CACNA1G, and FAT2 (P 0.01) (**Figure 1C**). To further explore the functional roles of these differentially expressed proteins, GSEA was conducted. The results indicated that differentially expressed proteins were mainly involved in processes such as visual system development, skin development, limb development, reactive oxygen species metabolic process, embryonic appendage morphogenesis, and embryonic limb morphogenesis (**Figure 1D**).

### Metabolomics analysis in the control and COVID-19 groups

**Figure 2:**
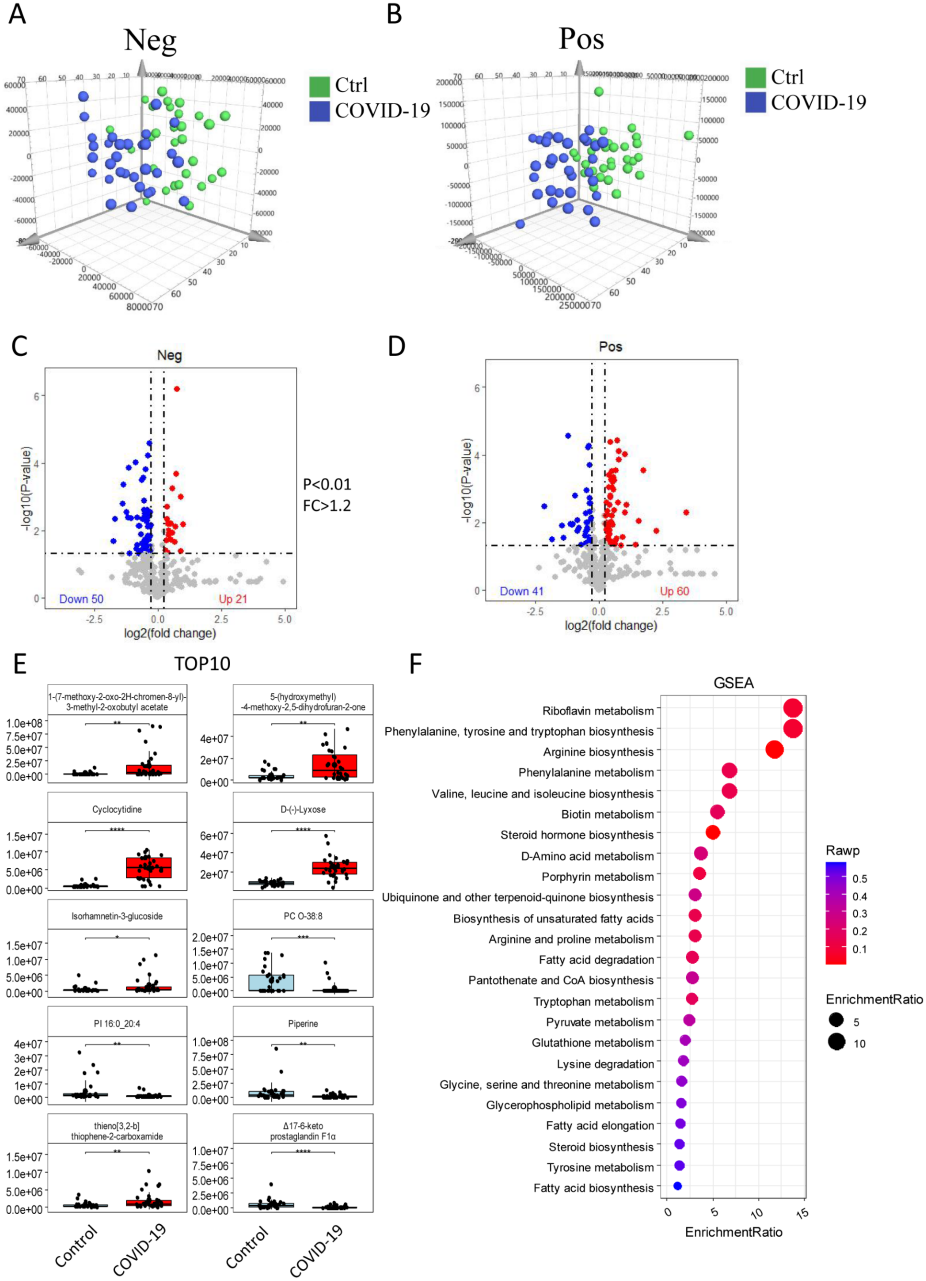
Serum metabolomics analysis. The differences of metabolites between the control and the COVID-19 groups were analyzed in both positive ion mode **(A)** and negative ion mode **(B)**. Differential expression is show in volcano plots **(C)** and **(D)** for negative and positive ion respectively. These show 172 differentially expressed metabolites, with 81up-regulated and 91 down-regulated. **(E)** The expression of the top 10 metabolites in the control and COVID-19 groups. **(F)** GSEA showing differential metabolites involved in key metabolic processes. The size of the circle indicates the number of enriched metabolites, while the red color reflects the statistical significance of the enrichment.

Serum metabolomics analysis, including both positive and negative ion mode data (**Figures 2A, B**), identified a total of 172 different metabolites, with 101 detected in positive ion mode and 71 in negative ion mode (**Figures 2C, D**). Compared with the control group, 81 metabolites were significantly increased in the COVID-19 group, including 60 in positive ion mode and 21 in negative ion mode. Conversely, 91 metabolites were significantly decreased, with 41 in positive ion mode and 50 in negative ion mode. The top 10 differential metabolites included 1-(7-methoxy-2-oxo-2H-chromen-8-yl)-3-methyl-2-oxobutylacetate, 5-(hydroxymethyl) -4-methoxy-2,5-dihydrofuran-2-one, cyclocytidine, D-(-)-Lyxose, isorhamnetin-3-glucoside, and thieno[3,2-b] thiophene-2-carboxamide, all of which were significantly elevated. On the other hand, PC O-38:8, PI16:0_20:4, piperine, and Δ17-6-keto prostaglandinF1α were significantly decreased (**Figure 2E**). Further GSEA on differential metabolites was conducted to explore the potentially affected metabolic pathways, and revealed disruptions in processes related to amino acid biosynthesis, amino acid metabolism, fatty acid metabolism, steroid and hormone metabolism. Notably, the riboflavin metabolism, the phenylalanine, tyrosine and tryptophan biosynthesis, and the arginine biosynthesis were significantly altered (**Figure 2F**).

### Integrating analysis of proteomics and metabolomics

**Figure 3:**
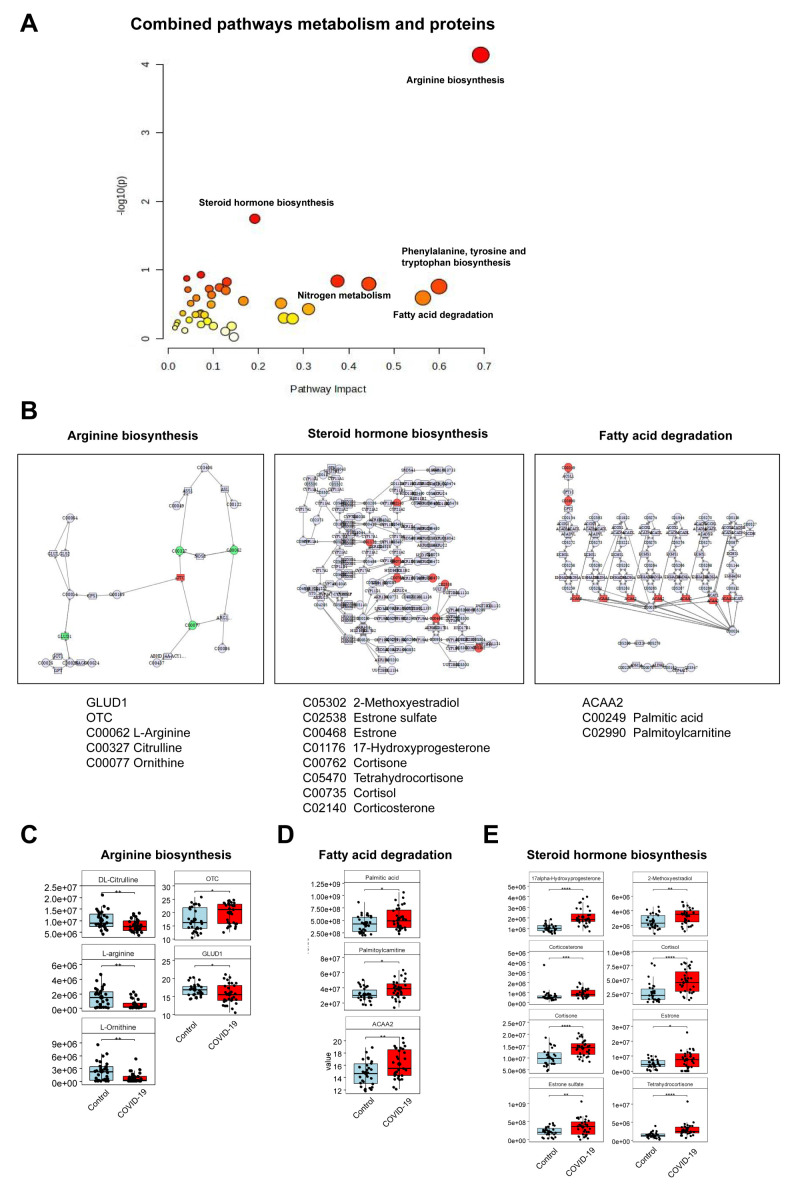
Combined analysis of proteomics and metabolomics. **(A)** Integration of metabolic pathways and protein analysis revealed that the differential metabolites affected key pathways. **(B)** Distinct metabolites significantly associated with arginine biosynthesis, steroid hormone biosynthesis, and fatty acid degradation pathways. **(C)** Three different metabolites and two abnormally expressed proteins involved in the arginine biosynthesis. **(D)** Two different metabolites and one abnormally expressed protein involved in the fatty acid degradation. **(E)** Eight up-regulated metabolites involved in the steroid hormone biosynthesis pathway.

Furthermore, we conducted an integrated analysis of differentially expressed proteins and metabolites, identifying the most enriched metabolic pathways: arginine biosynthesis, steroid hormone biosynthesis, nitrogen metabolism, and fatty acid degradation (**Figures 3A, B**). In the arginine biosynthesis pathway, three metabolites, DL-Citrulline, L-arginine, and L-ornithine were identified, along with two proteins, OTC and GLUD1, which were abnormally expressed (**Figure 3C**). The fatty acid degradation pathway revealed two different metabolites, plamitic acid and plamitoylcarnitine, and one differentially expressed protein, ACAA2 (**Figure 3D**). Eight differential metabolites, including 17alpha-Hydroxyprogesterone, 2-Methoxyestradiol, corticosterone, and cortisol, were up-regulated in the steroid hormone biosynthesis pathway (**Figure 3E**). Through the network analysis of the correlation between these pathways, this study found that arginine biosynthesis, steroid hormone biosynthesis, and fatty acid degradation pathways were most significantly affected in pregnant women infected with SARS-CoV-2 in the third trimester. The most significantly affected pathways and associated proteins/metabolites were summarized in **Table 4**.

**Table 4:** Summary of key differential pathways and molecules.

Pathway	Key differential proteins/metabolites	Regulation	P value
Arginine Biosynthesis	DL-Citrulline	Down	<0.01
	L-arginine	Down	<0.01
	L-Omithine	Down	<0.01
	OTC	Up	<0.05
	GLUD1	Up	<0.05
Fatty Acid Degradation	Plamitic acid	Up	<0.05
	Plamitoylcarnitine	Up	<0.05
	ACAA2	Up	<0.01
Steroid Hormone Biosynthesis	17alpha-Hydroxyprogesterone	Up	<0.0001
	2-Methoxyestradiol	Up	<0.01
	Corticosterone	Up	<0.001
	Cortisol	Up	<0.0001
	Cortisone	Up	<0.0001
	Estrone	Up	<0.05
	Estrone sulfate	Up	<0.01
	Tetrahy drocortisone	Up	<0.0001

## Discussion

The SARS-CoV-2 virus continues to evolve with frequent mutations, leading to recurrent infections. The maternal and infant outcomes of pregnant women infected with SARS-CoV-2 have attracted significant attention. In recent years, the pregnancy outcomes of SARS-CoV-2 infection have been discussed in many studies, but the specificity of the induced effect during pregnancy is still unclear. A large-scale cohort study by Jering et al. (18) demonstrated that pregnant women infected with SARS-CoV-2 face a significantly increased risk of preterm birth, stillbirth, myocardial infarction, and venous thromboembolism. Furthermore, research has shown that the incidence of preeclampsia in the SARS-CoV-2-infected group was 8.1%, markedly higher than the 4.4% observed in the non-infected group (19). However, studies on placental involvement in SARS-CoV-2 infection have detected viral presence in only a minority of cases, with no evidence of infection in the majority, suggesting a low risk of vertical transmission of COVID-19 (20, 21).

This study demonstrated that the incidences of cesarean section, postpartum reproductive tract infections, and fetal distress in the COVID-19 group were significantly higher compared to the control group. The elevated rate of fetal distress in pregnant women infected with SARS-CoV-2 during the third trimester may be attributed to the persistent fever, which led to increased basal metabolism, dehydration, and increased oxygen consumption ultimately disrupted the intrauterine environment of the fetus, contributing to fetal distress (22). Additionally, consistent with previous studies (23), our findings revealed that SARS-CoV-2 infection is associated with thrombocytopenia, as evidenced by a downward trend in platelet counts in the COVID-19 group. This condition likely exacerbates the risk of adverse pregnancy outcomes.

Our study identified the differential biomarkers between pregnant women infected with SARS-CoV-2 and healthy pregnant women in late phases of pregnancy. The results indicated that during the acute infection period of SARS-CoV-2, an acute inflammatory response was observed in pregnant women. Serum amyloid A proteins, specifically SAA1 and SAA2, significantly upregulated during the infection phase (24). SAA promotes inflammation by activating chemokines, and even at low concentrations, it can exacerbate the inflammatory response by enhancing chemotaxis (25). Previous studies have confirmed that SAA1 and SAA2 promote reactive oxygen species and mediate inflammatory processes. They can also regulate the invasion of trophoblast cells, a critical part of placenta development, by modulating metalloproteinase activity, thereby playing a vital role in placental formation and homeostasis (26).

Subsequent studies have shown that SAA proteins are closely associated with inflammatory biomarkers such as IL-6, D-Dimer, and high-sensitivity C-reactive protein (hsCRP), and can be used either independently or in combination with these markers as reliable prognostic indicators for COVID-19 (27). Furthermore, elevated levels of SAA may also increase the risk of spontaneous preterm birth (28). Building on previous findings, we hypothesize that elevated SAA levels may disrupt the invasion of trophoblast cells into the uterine decidua, potentially leading to adverse pregnancy outcomes. It is speculated that the expression levels of SAA1 and SAA2 have certain predictive value for adverse pregnancy outcomes of women infected with SARS-CoV-2 at the end of pregnancy. Early medical intervention to mitigate increases in SAA levels should be implemented promptly. However, the precise mechanisms underlying the role of SAA in pregnancy outcomes require further validation through additional clinical studies.

In the present study, we observed significant upregulation of the expression levels of both WDR19 and UHRF1BP1. Studies have demonstrated that the WDR19 protein plays a critical role in retinal development and function (29). Variants of the UHRF1BP1 protein are strongly associated with the pathogenesis of systemic lupus erythematosus, a condition often accompanied by skin lesions (30). On the other hand, down-regulated CACNA1G (31) and FAT2 (32) proteins are associated with abnormal cardiac function and various cancers, such as colorectal cancer, esophageal cancer and gastric cancer, respectively. GO pathway analysis revealed that the differentially expressed proteins between SARS-CoV-2 infected and uninfected pregnant women were predominantly involved in processes related to vision, skin and limb development, reactive oxygen species metabolism, embryonic appendage morphogenesis, and embryonic limb morphogenesis. These findings align with previous research, further underscoring the critical roles of these proteins in maintaining essential physiological functions.

Integrating analysis of proteomics and metabolomics revealed that the most significantly affected pathways were arginine biosynthesis, steroid hormone biosynthesis, and fatty acid degradation. Consistent with our findings, prior studies have reported alterations in metabolic pathways such as arginine biosynthesis, glutamate metabolism, and sphingolipid metabolism during SARS-CoV-2 acute infection (33, 34). L-arginine, as an essential amino acid, plays a crucial role in embryonic survival, fetal and neonatal growth, as well as in the maintenance of vascular tone and hemodynamics (35). L-arginine is a precursor for nitric oxide synthesis in the body, and nitric oxide is indispensable for the normal development of the placenta and fetal growth. Studies have shown that supplementation with L-arginine during pregnancy can significantly improve fetal growth, reduce maternal blood pressure, and prevent the occurrence of preeclampsia (36). Arginine has a wide range of functions in the body, serving not only as a key factor in immune system function but also playing a vital role in the normal function of the placenta and the growth and development of the fetus.

Steroid hormones, including glucocorticoids, androgens, and estrogens, exert profound effects on the immune system, with glucocorticoids particularly known for potent anti-inflammatory and immunosuppressive properties (37). Studies have shown that macrophages in COVID-19 patients exhibit functional impairments (38), which may explain the higher rate of postpartum reproductive tract infections in the COVID-19 group in this study. It is speculated that the body is still in a recovery period after SARS-CoV-2 infection, with an imbalance in the immune system. According to research, disturbances in fatty acid metabolism may lead to intrauterine growth restriction and negatively impact the placental nutrient exchange function, potentially resulting in fetal developmental delay and increasing the risk of postpartum complications (39). Additionally, a study on plasma proteomic and metabolomic profiles of COVID-19 survivors six months post-discharge found that multiple unsaturated fatty acid metabolites were down-regulated (40). This is consistent with the significant impact on fatty acid degradation observed in our study.

Moreover, differential proteins and metabolites showed that pathways related to visual development and riboflavin metabolism were most prominently affected. Particularly, the NLR in the COVID-19 group was significantly decreased in the third trimester of pregnancy compared with the control group. Considering this physiological index and the positive correlation between reduced NLR and retinopathy reported in previous studies (41–43), we hypothesize that SARS-CoV-2 infection may exert a substantial impact on fetal visual development by disrupting maternal immune balance. This hypothesis requires further validation through large-scale, prospective studies with long-term follow-up.

Our study is the first to systematically characterize the serum metabolite and protein expression profiles of women infected with SARS-CoV-2 at the end of pregnancy. These findings provide valuable data to deepen the understanding of the interactions between viral infection and the host. Furthermore, this study aids in evaluating the long-term effects of SARS-CoV-2 on maternal and infant health, providing important reference for the development of targeted interventions and protective measures.

However, this study has some limitations. Firstly, as this study was conducted at a single center with a limited sample size, potential selection bias may have been introduced. Although the sample size was sufficient for preliminary exploration, it may restrict the generalizability of the findings. Therefore, future studies are needed to involve multi-center and large-sample cohorts, incorporate independent validation cohorts, and further verify our findings through methods such as parallel reaction monitoring (PRM) and targeted metabolomics. Secondly, the COVID-19 vaccination status of pregnant women was not systematically collected, making it impossible to assess the potential impact of vaccination status on SARS-CoV-2 infection. The absence of follow-up data prevents us from assessing the long-term effects of these molecular changes, necessitating future studies with longer follow-up periods to validate our findings. Thirdly, we did not conduct related analyses on the genomics of pregnant women during the acute infection phase, which limits our ability to accurately evaluate whether these changes are hereditary. Moreover, psychological factors such as stress, anxiety, and depression were not systematically assessed in this study. Future research will incorporate standardized assessments to better control for these potential confounders.

Future research will aim to expand the collection and preparation of biological specimens from SARS-CoV-2 infection in other stages of pregnancy (first and second trimesters). A dynamic monitoring approach will be employed to examine biochemical indicators, serum metabolites, and specific proteins profiles across all stages of pregnancy using serum protein-metabolomics technology. By integrating clinical data and follow-up data, the effects of SARS-CoV-2 on pregnant women and the long-term development of their offspring (0–3 years old) can be systematically evaluated. Furthermore, combining machine learning algorithms with large-cohort validation may enable the identification of predictive biomarkers for specific adverse pregnancy outcomes. From a public health perspective, during pandemics, routine biochemical monitoring and early screening (e.g., SAA, NLR) for infected pregnant women are recommended to identify individuals at higher risk. Based on observed biochemical and metabolic abnormalities, the development of personalized early intervention strategies may help reduce the risk of adverse pregnancy outcomes and improve maternal and fetal health outcomes.

In conclusion, this is the first study to investigate the effects of SARS-CoV-2 infection on the serum proteomic and metabolomic profiles of pregnant women in the third trimester. Our findings indicated that acute SARS-CoV-2 infection significantly disrupts key metabolic pathways, including arginine biosynthesis, steroid hormone biosynthesis, and fatty acid degradation. The infection also impacts processes critical for fetal development, such as visual, skin and limb development, reactive oxygen species metabolism, embryonic appendage morphogenesis, and embryonic limb morphogenesis, with visual development being particularly affected. To comprehensively assess the long-term effects of SARS-CoV-2 on maternal and infant health, prolonged follow-up studies of infected pregnant women are essential. These studies should have monitored the health of offspring, including growth and development, immune system development, psychological behavior, and other aspects.

## Data Availability

The raw data supporting the conclusions of this article will be made available by the authors, without undue reservation.

## Supplementary material

Supplementary Figure 1 Quality control analysis of plasma proteomics data. **(A)** Heatmap of Pearson correlation between biological replicates, showing good biological reproducibility (r=0.80-0.99) between most sample pairs. **(B)** PCA plot based on all experimental and QC samples, showing tight clustering of QC samples, indicating good methodological stability.

Supplementary Figure 2 Workflow of the integrated multi-omics analysis.

Supplementary Table 1 Clinical features and treatments of the COVID-19 group.
