# Supplementary figures and images for: Proteomic and metabolomic analysis of serum in women infected with COVID-19 during late pregnancy
Source: Front Immunol. 2025 Jun 11;16:1589239. doi: 10.3389/fimmu.2025.1589239 (PMC12187731; doi:10.3389/fimmu.2025.1589239)

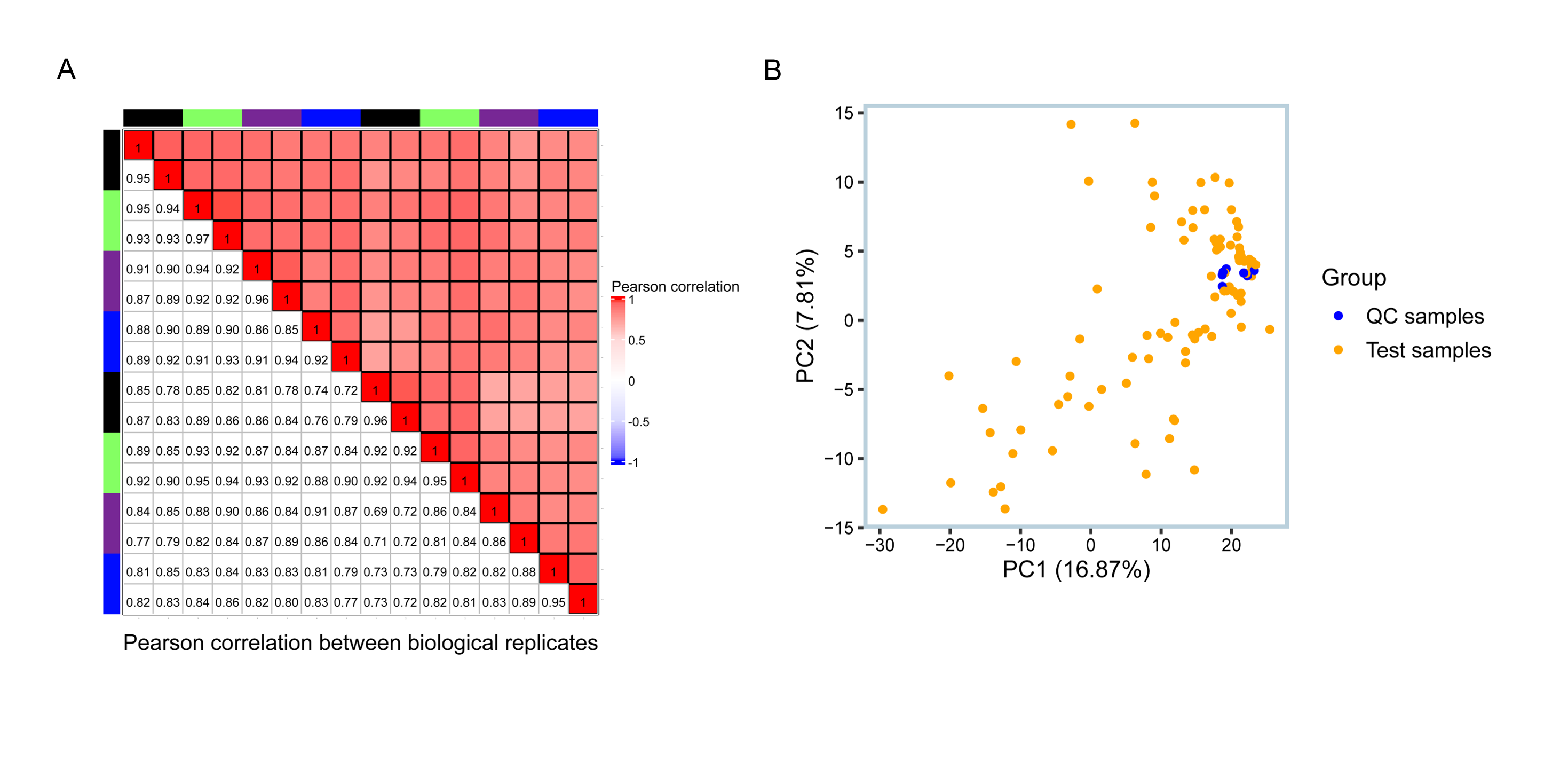

Supplement: Supplementary Figure 1 — Quality control analysis of plasma proteomics data. (A) Heatmap of Pearson correlation between biological replicates, showing good biological reproducibility (r=0.80-0.99) between most sample pairs. (B) PCA plot based on all experimental and QC samples, showing tight clustering of QC samples, indicating good methodological stability. [file Image1.tif]

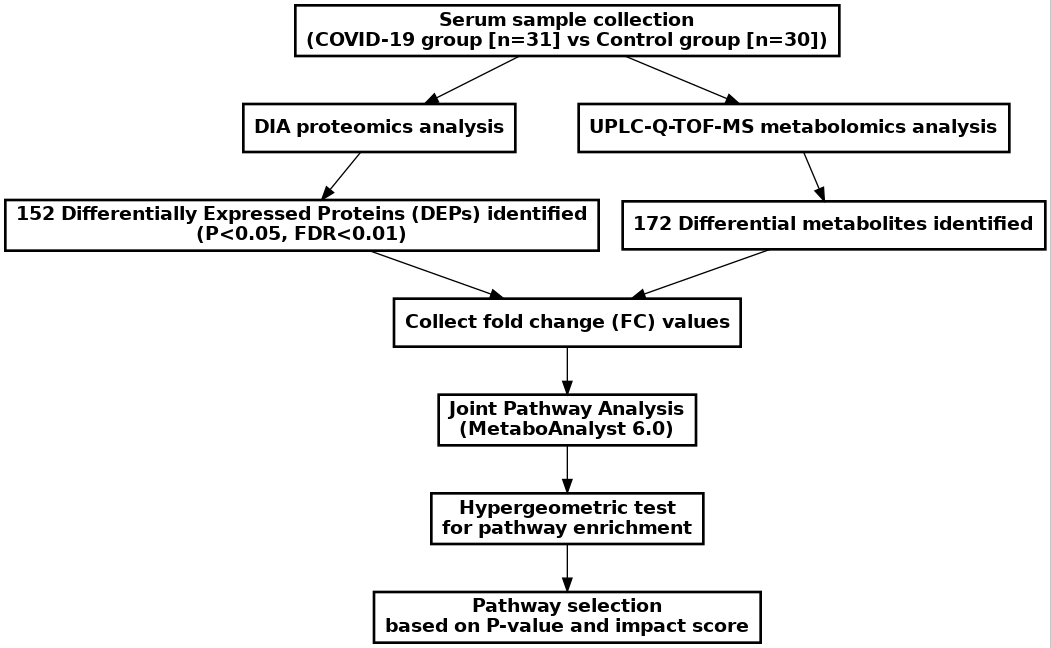

Supplement: Supplementary Figure 2 — Workflow of the integrated multi-omics analysis. [file Image2.tif]
