# Supplementary material for: Proteomic and metabolomic analysis of serum in women infected with COVID-19 during late pregnancy
Source: Front Immunol. 2025 Jun 11;16:1589239. doi: 10.3389/fimmu.2025.1589239 (PMC12187731; doi:10.3389/fimmu.2025.1589239)
Supplement: Supplementary Table 1 — Clinical features and treatments of the COVID-19 group. [file Table1.docx]

**Supplementary Table 1.** Clinical features and treatments of the COVID-19 group

| **ID** | **WHO Severity Classification** | **Maximum Temperature (℃)** | **Main Symptoms** | **Main Treatment** |
| --- | --- | --- | --- | --- |
| 1 | Mild | 37.5 | Fever | Lianhua Qingwen Capsule |
| 2 | Moderate | 39.5 | Fever, Dizziness, Sore throat, Muscle soreness | Acetaminophen |
| 3 | Mild | 37.6 | Fever | - |
| 4 | Moderate | 38.3 | Fever, Dizziness, Chest distress | Ibuprofen |
| 5 | Mild | 38.5 | Fever | Temperature monitoring |
| 6 | Moderate | 37.9 | Fever, Runny Nose, Fatigue, Muscle soreness | - |
| 7 | Asymptomatic | - | - | - |
| 8 | Mild | 38.5 | Fever | Temperature monitoring |
| 9 | Moderate | 38.2 | Fever, Sore throat, Cough with sputum | Acetaminophen |
| 10 | Moderate | 38.5 | Fever, Sore throat, Fatigue, Muscle soreness | Acetaminophen, Pudilan Anti-inflammatory Liquid |
| 11 | Asymptomatic | - | - | - |
| 12 | Moderate | 38.2 | Fever, Headache, Chills | Temperature monitoring |
| 13 | Moderate | 38.0 | Fever, Fatigue, Sore throat, Muscle soreness | Temperature monitoring |
| 14 | Moderate | 38.1 | Fever, Muscle soreness, Headache | Temperature monitoring |
| 15 | Moderate | 38.6 | Fever, Nasal obstruction, Runny Nose, Sore throat, Muscle soreness, | Acetaminophen |
| 16 | Moderate | 38.9 | Fever, Headache, Muscle soreness, Fatigue, Runny Nose | Acetaminophen |
| 17 | Moderate | 38.0 | Fever, Sore throat | Temperature monitoring |
| 18 | Mild | 38.2 | Fever, Cough | Acetaminophen |
| 19 | Mild | 38.3 | Fever, Chills | Acetaminophen |
| 20 | Moderate | 38.3 | Fever, Dry cough, Fatigue | Temperature monitoring |
| 21 | Mild | 38.3 | Fever, Cough | Acetaminophen |
| 22 | Moderate | 38.9 | Fever, Headache, Dizziness, Productive cough with yellow sputum | Acetaminophen, Chuanbei Pipa Syrup, Budesonide, Terbutaline |
| 23 | Moderate | 37.6 | Fever, Cough with sputum | Temperature monitoring |
| 24 | Moderate | 38.3 | Fever, Chills, Cough | Minor Bupleurum Granules |
| 25 | Moderate | 38.8 | Fever, Headache, Fever, Chills, Sore throat, Nasal obstruction | Ibuprofen |
| 26 | Moderate | 38.0 | Fever, Sore throat, Dizziness, Fatigue, Muscle soreness | Siji Antiviral Oral Liquid |
| 27 | Moderate | 38.6 | Fever, Sore throat, Cough with sputum | Yin Hua Soft Capsules, Eucalyptol-Limonene-Pinene Enteric Soft Capsules, Ibuprofen |
| 28 | Moderate | 37.6 | Fever, Cough with sputum, Headache, | Fei Li Ke Mixture |
| 29 | Moderate | 39.0 | Fever, Sore throat, Cough | Ibuprofen |
| 30 | Moderate | 39.2 | Fever, Productive cough with yellow sputum | Paracetamol, Pseudoephedrine and Dextromethorphan for Suspension, Chuanbei Pipa Syrup |
| 31 | Moderate | 38.5 | Fever, Dry mouth and tongue, Sore throat | Temperature monitoring |
